# Accept Anxiety to Improve Sleep: The Impact of the COVID-19 Lockdown on the Relationships between Mindfulness, Distress, and Sleep Quality

**DOI:** 10.3390/ijerph182413149

**Published:** 2021-12-13

**Authors:** Marco Mirolli, Luca Simione, Monica Martoni, Marco Fabbri

**Affiliations:** 1 Institute of Cognitive Sciences and Technologies, National Research Council (ISTC-CNR), Via San Martino della Battaglia 44, 00185 Rome, Italy; luca.simione@istc.cnr.it; 2 Department of Experimental, Diagnostic and Specialized Medicine, St. Orsola-Malpighi Hospital, University of Bologna, Via Massarenti 9, 40138 Bologna, Italy; monica.martoni@unibo.it; 3 Department of Psychology, University of Campania Luigi Vanvitelli, Viale Ellittico 31, 81100 Caserta, Italy

**Keywords:** sleep quality, mindfulness, distress, COVID-19 lockdown, longitudinal study, path analysis

## Abstract

It has been recently proposed that mindfulness can improve sleep quality through the mediating role on psychological distress and that acceptance may play a pivotal role in mindfulness beneficial effects. The aim of the present work was to understand the effects of the COVID-19 lockdown on dispositional mindfulness, sleep, and distress, and on their relationships. In particular, we wanted to test the hypothesis that the detrimental effects of lockdown on sleep depended on mindfulness and distress (including anxiety and depression) and that the acceptance facet of mindfulness played the leading role. A longitudinal study based on self-report questionnaires was conducted on 39 Italian adults (M age = 35.03, SD = 14.02; 21 men) assessing mindfulness, distress, and sleep quality before (23 December 2019–8 March 2020) and during (27 April 2020–10 May 2020) the first Italian COVID-19 lockdown. Lockdown decreased mindfulness while increasing distress and sleep problems. Path analysis showed that the effects of lockdown on sleep were fully mediated by mindfulness and distress. Furthermore, a more detailed analysis showed that these effects were mainly dependent on the acceptance component of mindfulness working through anxiety. The present study confirms, in the context of the COVID-19 lockdown, a model according to which mindfulness, and specifically acceptance, influences sleep through the mediating role of distress.

## 1. Introduction

The spread of COVID-19 resulted in a high prevalence of sleep problems not only in COVID-19 patients, but also in healthcare workers and in the general population [1,2]. In Italy, the first lockdown, which involved home confinement and social distancing for the entire population from 10 March to 3 May 2020, affected both sleep and mental health, with an increase in sleep difficulties, especially in people with a higher level of depression, anxiety, and stress [3,4]. Indeed, both the pandemic itself and the resulting quarantine have been shown to increase stress and stress-related disturbances [1,5,6,7]. However, little is known about the mechanisms underlying these deleterious effects.

A possible psychological factor that is likely to be relevant is mindfulness, which can be defined as being present in the moment intentionally and with a non-judging attitude [8]. Mindfulness has been associated with better sleep quality [9], greater well-being [10], and lower levels of depression and anxiety [11], and mindfulness-based approaches have been used to improve insomnia, depression, and anxiety symptoms [12]. Importantly, mindfulness has been shown to have a protective effect on sleep during the COVID-19 lockdown [13]. Recently, Simione et al. [14] have shown that the positive relationships between dispositional mindfulness and sleep quality fully depend on the mediational role of stress, which is in accordance with recent models of insomnia such as the stress-diathesis [15] and the metacognitive [16] models.

However, mindfulness is a multidimensional concept [17], and different mindfulness components have different effects on different outcomes [18]. Lindsay and Creswell [19] proposed the Monitoring and Acceptance theory (MAT), according to which mindfulness works through the two mechanisms of attention monitoring and acceptance: while monitoring alone tends to increase affective reactivity, monitoring and acceptance together lead to increased psychophysical well-being. However, Simione et al. [20] have shown that the beneficial effects of mindfulness seem to depend almost entirely on acceptance alone, with monitoring playing a deleterious role in only a few cases, which, interestingly, include sleep problems.

In the present longitudinal study, we assessed dispositional mindfulness, distress symptoms, and sleep problems in the same sample both before and during the first Italian COVID-19 lockdown. While predicting that lockdown would worsen both distress symptoms and sleep problems, we were interested in testing whether mindfulness, and specifically its acceptance component, could play a mediating role in these changes. In particular, on the basis of the reviewed literature, we hypothesized that lockdown may increase sleep problems by reducing mindfulness and increasing psychological distress and that the beneficial effects of mindfulness would depend mainly on acceptance.

## 2. Materials and Methods

### 2.1. Participants and Procedure

A convenience sample method was used, recruiting participants from the general population through email and social media (no mindfulness practice nor any particular interest in mindfulness was required for participation). During the period from 23 December 2019 to 8 March 2020, 43 volunteers participated in the survey, after reading the written consent form and explicitly agreeing to take part in the study. In this first period, all questionnaires (see below) were individually administered in paper-and-pencil form. The study protocol was approved by the Ethics Committee of the Department of Psychology at the University of Campania Luigi Vanvitelli and it originally aimed to investigate the relationship between dispositional mindfulness and different psychological and cognitive variables. When home restriction was adopted in Italy in response of the COVID-19 pandemic, the aim of the study was changed in order to assess the effect of lockdown on sleep, mindfulness, and distress. Consequently, we re-contacted all participants and asked them to complete an online survey (using the Google Moduli platform) including the same questionnaires filled in during the first period. Thirty-nine of the 43 volunteers responded and filled the questionnaires from 17 April to 10 May, 2020. We collected the following demographic data for each participant: age, sex, city, educational level, and occupation. The mean age was 35.03 years (SD = 14.02 years), with 21 men. All participants lived in the south of Italy, the educational level ranged from eight grade diploma to PhD title, and the occupational status covered unemployment, student, and workers in both private and public fields.

### 2.2. Materials

Dispositional mindfulness was measured using the Italian version of the Five Facets Mindfulness Questionnaire (FFMQ [21]), containing 39 items divided in five subscales: observing, describing, acting with awareness, non-judging, and non-reacting. Participants were requested to rate each statement on a 5-point Likert scale. Higher total scores indicate higher dispositional mindfulness. Following the literature on MAT theory [19,20], we considered monitoring as being represented by observing and acceptance as being represented by non-judging and non-reacting. The psychometric properties of this scale are good [21].

The Italian version of the Hospital Anxiety and Depression Scale (HADS [22]) was administered to assess general distress. The HADS consists of 14 items divided in two subscales: anxiety and depression. Participants are requested to rate how they have been feeling in the past week on a 4-point scale. The psychometric properties of the HADS are good [22]. Following Iani et al. [22], the total score was used as a measure of general psychological distress.

In order to detect sleep quality as well as sleep-related wake disorders, the Italian version of the Mini Sleep Questionnaire (MSQ [23]) was used. In this questionnaire, 6 of the 10 items are related to sleep problems while 4 items are related to wake problems. Respondents had to indicate the frequency of occurrence for each statement in the past week on a 7-point scale. Beyond the total score, as suggested by Natale et al. [23], we also calculated the scores of the sleep and wake subscales. The psychometric properties of the MSQ are good [24].

Given that circadian typology has been shown to influence sleep problems [25], in order to control for such a factor, we also administered the Italian version of the reduced Morningness-Eveningness Questionnaire to measure circadian typology (rMEQ [26]). The rMEQ includes 5 items and the total score is obtained by summing up all the items, with higher scores reflecting a morningness preference. The psychometric properties of rMEQ are good [26].

### 2.3. Data Analysis

First, we checked for the presence of a common method bias in the responses using two tests: the Harman’s one-factor test and the correlation matrix test [27]. In the first test we computed the variance explained by a single-factor exploratory model including all the items administered, considering the bias to be present if the proportion of variance explained by this single factor was higher than 50%. In the second test we considered the correlation matrix between all assessed variables, considering the bias to be present if correlations were higher than 0.90. In both tests, for each variable we considered all the observations (i.e., each participant assessed both before and during the lockdown).

Secondly, we assessed the effect of lockdown on the measured variables through a repeated-measures MANOVA followed by a series of one-way repeated-measures ANCOVAs to assess the effect of time on each dependent variable, while controlling for the effects of sex, age, and education level. As a measure of effect size, we used the partial eta squared which is recommended in order to improve the comparability of effect sizes between studies [28].

Then, we tested our hypothesis that lockdown onset impacted mindfulness, which affected psychological distress, in turn influencing sleep problems. To this aim, we tested the indirect effect of mindfulness on the effect of time on distress/sleep by using path analysis with the Huber-White robust standard errors estimator and bias-corrected confidence intervals that test indirect or mediated effects [29]. In particular, we tested two models. In the first one we included the total score for each scale, in order to assess the relationships between mindfulness, general distress, and sleep problems. In the second model, we used the subscale scores of each questionnaire to investigate the differential contribution of each facet or aspect to the considered effects. Regarding mindfulness, following the literature on MAT theory [19,20], we took into account only the variables considered to be related to either monitoring (i.e., observing) or acceptance (i.e., non-judging and non-reacting). In both models, we controlled for the effects of age, sex, education level, and chronotype (rMEQ score). 

As both models were fully saturated (i.e., they perfectly fitted the data because they had as many parameters as there were values to be fitted) no goodness of fit scores could be calculated. In order to both obtain interpretable goodness of fit statistics and reduce the number of free parameters so to counterbalance the small numerosity of the sample, we also analyzed simplified versions of the models where all non-significant path (and covariates) were removed. For each model, we calculated the following fitting indexes: χ2 statistics, comparative fit index (CFI), Tucker–Lewis index (TLI), root mean square error of approximation (RMSEA), and standard root mean square residual (SRMR). Model fit was considered as adequate with the following values: non-significant χ2, CFI and TLI above 0.95, RMSEA of 0.06 or less, SRMR of 0.08 or less [30]. Raw data are available as Supplementary Materials.

## 3. Results

Both tests for a common method bias showed that no such bias was present. The variance explained by a single-factor model was only 14.72%, much lower than the threshold of 50% (Harman’s one-factor test). Furthermore, all the correlation coefficients between our variables were between 0.01 and 0.65 (absolute value), that is smaller than the threshold value of 0.90 (correlation matrix test).

### 3.1. Effects of Lockdown

The MANOVA assessing the effect of lockdown on our variables was significant for time, Pillai’s Trace = 0.57, *F*(7,38) = 6.04, *p* < 0.01, indicating a significant overall impact of lockdown on the variables. The results of the subsequent one-way repeated-measures ANCOVAs are reported in Table 1 (together with Cronbach’s αs of all variables). Two mindfulness facets (i.e., observing and non-judging) and the total mindfulness score changed as a function of time. In particular, observing increased during lockdown while non-judging and total mindfulness decreased. Regarding psychological distress, anxiety did not change, while both depression and HADS total increased. Regarding sleep-related problems, sleep quality (sleep factor) decreased, while daytime sleepiness (wake factor) and MSQ total did not change. Lastly, circadian typology (rMEQ) did not change. Overall, these results showed that lockdown impacted different areas of psychological functioning, including mindfulness, distress, and sleep quality. 

### 3.2. From Lockdown to Sleep Problems through Mindfulness and Distress

**Figure 1:**
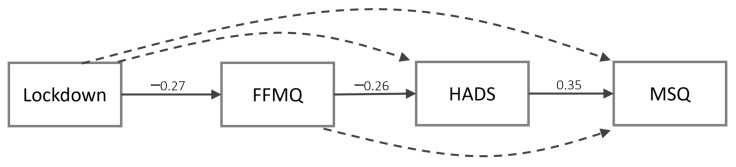
First model including only the total scores. Continuous arrows represent significant paths, while dotted arrows represent non-significant paths. Standardized coefficients are reported only for significant paths.

Table 2 shows the estimated coefficients of the model including the high-level variables (Figure 1). This analysis revealed that lockdown significantly decreased mindfulness, and this, in turn, decreased distress, while distress increased sleep problems. Furthermore, we found three significant indirect effects: from lockdown to distress through mindfulness (b = 0.61, CI = [0.08, 1.64], SE = 0.35, β = 0.07), from lockdown to sleep through both mindfulness and distress (b = 0.51, CI = [0.05, 1.88], SE = 0.36, β = 0.03), and from mindfulness to sleep problems through distress (b = −0.06, CI = [−0.16, −0.01], β = −0.09). All indirect effects represented full mediation, as time did not significantly affect distress or sleep problems, nor did mindfulness directly affect sleep. Testing the model while removing the covariates did not significantly alter any of the considered paths. Hence, we tested the fit of the simplified model containing only significant paths and no covariate. This showed good fit statistics: χ2(3) = 1.04, *p* = 0.79, CFI = 1.00, TLI = 1.00, RMSEA = 0.01, SRMR = 0.03.

### 3.3. The Effects of Mindfulness Depend on Acceptance

**Figure 2:**
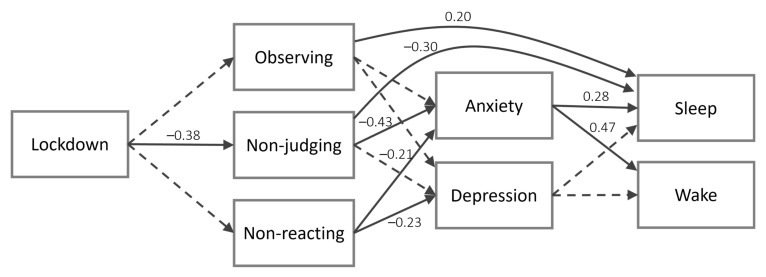
Second model including all the subscale scores. Continuous arrows represent significant paths, while dotted arrows represent non-significant paths. Standardized coefficients are reported only for significant paths. For the sake of clarity, only the direct paths from lower levels to higher levels that were significant are shown: e.g., the paths from lockdown to distress and sleep variables are not shown because they were not significant.

Table 3 shows the estimated coefficients of the model including the low-level variables (Figure 2). We found a significant effect of lockdown on non-judging but not on observing nor on non-reacting. Anxiety was significantly reduced by both non-judging and non-reacting but was not influenced by observing. Depression was not predicted by observing nor by non-judging, but it was reduced by non-reacting. Lastly, anxiety increased both components of the sleep-wake cycle (sleep and wake), whereas depression did not influence any of them. Regarding indirect effects, we found a significant path from lockdown to anxiety through non-judging (b = 1.01, CI = [0.36, 1.86], SE = 0.37, β = 0.17), two significant paths from non-judging to sleep (b = −0.12, CI = [−0.29, −0.02], SE = 0.07, β = −0.12) and wake (b = −0.17, CI = [−0.34, −0.06], SE = 0.07, β = −0.20) through anxiety, and two significant paths from lockdown to both sleep (b = 0.55, CI = [0.04, 1.47], SE = 0.36, β = 0.05) and wake (b = 0.82, CI = [0.21, 1.81], SE = 0.40, β = 0.08) through non-judging and anxiety. Lockdown had no significant direct effect on anxiety, depression, sleep, or wake. However, both observing and non-judging had a significant direct effect on sleep (the first positive, the second negative). To sum up, this model confirmed that the main direct and indirect effects of time were mostly dependent on acceptance (in particular, on the non-judging facet). Observing had a direct deleterious effect only on sleep, while non-reacting reduced both anxiety and depression; none of these effects, however, were influenced by time. Even in this case, testing the model while removing the covariates did not significantly alter the results. The simplified model including only significant paths and no covariates revealed acceptable fit statistics: χ2(10) = 9.89, *p* = 0.45, CFI = 1.00, TLI = 1.00, RMSEA = 0.01, SRMR = 0.09.

## 4. Discussion

The present study aimed to explain how the COVID-19 restrictions impacted on the sleep through an analysis of the mediational role of mindfulness and distress. Our results showed that the lockdown resulted in a general decrease in mindfulness (with an increase in observing and a decrease in non-judging), an increase in depression and distress, and an increase in sleep problems. Our first model fully supported our hypothesis that the effect of lockdown on sleep depended on mindfulness and distress. In particular, the model showed that lockdown decreased mindfulness, mindfulness decreased distress, and distress increased sleep problems. Furthermore, indirect pathways showed that mindfulness fully mediated the relationship between lockdown and distress, mindfulness and distress fully mediated the relationship between lockdown and sleep, and distress fully mediated the relationship between mindfulness and sleep. The second model supported the hypothesis that acceptance played the main role in the beneficial effects of mindfulness on sleep. In particular, it showed that: lockdown reduced non-judging; both acceptance facets (i.e., non-judging and non-reacting) decreased anxiety; non-reacting reduced depression; anxiety increased both components of the sleep-wake cycle. The only significant influence of the monitoring factor (i.e., observing) was an increase in sleep problems (which were also decreased by non-judging). Furthermore, indirect effects confirmed both the pivotal role of acceptance (and specifically of non-judging) in the beneficial outcomes of mindfulness and the mediated nature of the effect of lockdown on sleep: non-judging fully mediated the relationship between lockdown and anxiety, anxiety mediated the relationship between non-judging and problems in sleep (partially) and wake (fully), and non-judging and anxiety fully mediated the relationship between lockdown and both sleep and wake problems.

Several lines of research support the view that the effects of lockdown on sleep depend on the mediating role of mindfulness and distress. First, mindfulness is negatively correlated to stress [11,31] and mindfulness interventions have positive effects on stress and stress-related disorders [32]. Second, stress is well-known to have a deleterious effect on sleep [33], which is in accordance with the stress diathesis model of insomnia, according to which sleep problems depend mainly on stressful events and stress-induced cognitive intrusions [15]. Third, the mediational role of stress and stress-related disturbances in the link between mindfulness and sleep is supported by several cross-sectional studies [14,34] and is also in accordance with the meta-cognitive model of insomnia [16]: according to this model mindfulness can improve insomnia by reducing the distress produced by sleep-related worries, which are the main causes of the secondary arousal that contributes to insomnia. Furthermore, Simione et al. [14] have proposed that mindfulness could act on insomnia also by reducing primary arousal through a reduction of the impact of stressful events. Finally, a recent work involving two studies (one in Wuhan, China, and the other in the United Kingdom) demonstrated the protective role of mindfulness in the relationship between COVID-19-related stressors and decreases in sleep duration [13].

As far as mindfulness facets are concerned, the monitoring and acceptance components of mindfulness behaved in an opposite way: while non-judging decreased during lockdown, observing increased, and while acceptance facets (non-judging and non-reacting) jointly had beneficial direct and indirect effects on all distress and sleep variables, the monitoring facet (observing) had a deleterious effect only on sleep problems. The differential effect of lockdown on the two relevant mindfulness facets seems logical. It is reasonable that during the lockdown people tended to be more vigilant with respect to themselves and the surroundings due to the threat of illness, which might explain the higher observing scores. The same heightened perceived risk might also explain the decrease in non-judging, as the judgement of one’s thoughts and behaviors was considered to be important (and socially reinforced) for protecting one’s safety. Even the effects of these changes in mindfulness aspects on distress and sleep make sense given the pandemic context. Indeed, while these changes might be the result of trying to preserve one’s health, they had a detrimental effect on one’s well-being: they led to more anxiety (e.g., noticing more things to be worried about, worrying more about the health and well-being of oneself and loved ones), which in turn detrimentally impacted sleep.

Beyond being understandable given the very peculiar pandemic context, these results are also consistent with previous research. For example, acceptance has been associated with many beneficial outcomes including lower stress, anxiety, and depression [35], while a recent meta-analysis showed that observing correlates with a few psychological symptoms, including anxiety [18]. Consistently with the current results, in Simione et al. [20], sleep problems were the only outcomes (apart from general distress) that were predicted by the observing facet. According to the influential MAT theory of mindfulness, monitoring alone tends to increase affective reactivity, which can lead to both more psychological symptoms and a greater level of well-being, while acceptance moderates the effect of monitoring in a such way that together they lead to increased psychological well-being [19]. However, on the basis of both their own data and the available literature, Simione et al. [20] showed that these hypotheses were not well-supported, as monitoring was related to only a few psychological outcomes (mainly negative) while acceptance only rarely moderated monitoring, and, even when it did, it protected against the negative effects of monitoring rather than leading to the best psychological outcomes. For these reasons, the authors proposed an alternative hypothesis according to which acceptance alone is mainly responsible for the benefits of mindfulness, whereas monitoring plays only an ancillary role in developing acceptance, while sometimes providing negative consequences. Even though in the present study we could not test for the interaction between acceptance and monitoring due to our small sample size, our results seem to support this alternative hypothesis, as monitoring (observing) played a very limited deleterious role, while acceptance facets (non-reacting and especially non-judging) were the main drivers of change.

Shallcross et al. [36] proposed that mindfulness improves sleep through the mechanisms of experiential awareness, attentional control, and acceptance, which collectively target all the processes that contribute to sleep disturbance: rumination, primary arousal, secondary arousal, sleep monitoring/selective attention and effort, and distorted perceptions regarding sleep impairment. According to this view, acceptance works only on the last three factors, while the first two are targeted only by experiential awareness and attentional control. However, in our data acceptance alone was responsible for the benefits of mindfulness on sleep, in particular through a mediated effect on anxiety. Indeed, while experiential awareness and attentional control without acceptance may even be detrimental in case the current state is unpleasant and unwanted (e.g., stressful thoughts and lack of sleep), thus increasing rumination and primary arousal, acceptance has been associated with less worry and rumination [37], and with less stress and fewer stress-related disturbances [35]. Hence, it is likely that acceptance alone could act on all the processes that contribute to sleep problems.

Finally, we showed that the lockdown-related sleep problems depended on a decrease in mindfulness traits, and thus the present research adds evidence to the mounting literature recommending the use of mindfulness-based interventions to treat insomnia and sleep disturbances [12,16,36]. Furthermore, by showing the pivotal role of acceptance (non-judging) in linking lockdown and sleep problems, our results suggest that it may be interesting to design mindfulness-based interventions that focus particularly on developing acceptance skills so as to test their capacity to prevent sleep problems, particularly in stressful situations.

An important strength of the present study consists in being one of the few studies with “real” pre-lockdown measures of analyzed variables, thus leading to an authentic longitudinal study assessing the impact of the lockdown. Due to the impossibility of foreseeing the advent of the pandemic and the related restrictions, the majority of the previous studies concerning the effects of the pandemic on sleep had to make important compromises, which inevitably limited the reliability. For example, Cellini et al. [3] asked participants to think about the week before any restriction in Italy, which may introduce memory biases in subjects’ responses. Similarly, Salfi et al. [4] longitudinally assessed sleep quality, insomnia symptoms, and general distress (anxiety, depression, and stress) in an Italian sample from the first to the second wave of COVID-19 thus comparing similar situations, as the pandemic was continuously present in Italy between the two waves (with different degrees of risk).

However, the present study has its own limitations. First and foremost, the main limit of the present study lies in the small numerosity of the sample which was due to the fact that when the lockdown began, only a small group of participants had compiled the questionnaires. When evaluating model’s generalization, one should consider several factors, including the study design and the strength of path coefficients. Our study uses a longitudinal design, which is far more robust than a cross-sectional one, and our main direct and meditated paths reported medium sized effects (ranging from 0.25 to 0.47). As suggested in [38], to find a reliable medium-sized mediation path in a longitudinal study like our own with bootstrapping coefficients, about 40/50 participants should be sufficient. Moreover, our model could be considered unbiased as we did not face non-convergence and improper solutions problems during model estimation [39]. So, from this point of view, the numerosity of our sample was almost acceptable. However, our two models contained, respectively, 21 and 63 free parameters. Considering the rule of thumb requiring a 10:1 ratio between observations and free parameters [40], the numerosity of our sample was indeed too small. For this reason (and also to get non-identified models for which we could obtain interpretable goodness of fit statistics), we simplified our models by removing all non-significant paths and covariates, as testing both models while removing the covariates did not alter significantly any of the considered paths. In this way, we obtained two models whose paths were supported both by the literature and by the previous ‘full’ models. These ‘simplified’ models had, respectively, six and 12 free parameters, and both demonstrated good fit indexes. This makes the numerosity of our observation (78) adequate for the first model, while a bit too low for the second model, which consequently should be considered with more caution. Anyway, we think that the limitation due to the small sample was counterbalanced by the possibility of giving a real picture of the effects of lockdown restrictions on the assessed variables. Furthermore, the fact that our results confirmed both our hypotheses, which were based on the previous literature, suggests that the study power was enough for detecting at least the main true effects. Another limit of the present work depends on the measurement tool used for assessing mindfulness. Even if the FFMQ is the most widely used tool adopted for measuring mindfulness, the acceptance dimension is defined by two distinct measures (non-judging and non-reacting), which could be a source of confusion. Future research should confirm the role of acceptance in protecting from sleep problems using another mindfulness tool such as the Philadelphia Mindfulness Scale (PHLMS [41]), as this includes only one scale for acceptance and one for awareness (which can be considered as a measure of attention monitoring). Finally, our study used only self-report questionnaires, which could limit the reliability and validity of our findings due to well-known problems related to self-report measures, such as limited introspective abilities, problems of interpretation, and response biases such as social desirability. Future studies could improve this aspect by also adopting more objective measures of the assessed variables. From this point of view, the development of behavioral measures of mindfulness represents an important challenge for future research [42].

## 5. Conclusions

The present longitudinal work showed that the detrimental effect of the first Italian COVID-19 lockdown on sleep was fully mediated by mindfulness and distress and that these effects were dependent on the acceptance component working through anxiety, thus confirming our hypotheses based on previously published cross-sectional results [14,20]. By significantly advancing our knowledge of the mechanisms linking sleep to mindfulness and distress, this work not only adds evidence to the mounting literature recommending the use of mindfulness-based interventions to treat insomnia and sleep disturbances, but it also suggests the possibility to develop novel mindfulness-based interventions that focus particularly on acceptance for preventing sleep problems in stressful situations.

## Figures and Tables

**Table 1:** Reliability, means, standard deviations, and one-way ANCOVA statistics for variables measured before (Time 0) and during (Time 1) lockdown.

			Time 0		Time 1			
Scale	Variable	Cronbach’s α	M	SD	M	SD	*F*(1,37)	η^2^_p_
FFMQ	**Observing**	**0.79**	**27.36**	**6.7**	**29.74**	**4.56**	6.51 *	**0.15**
	**Non-judging**	**0.80**	**27.18**	**5.4**	**22.49**	**5.57**	16.56 **	**0.31**
	Non-reacting	0.71	21.49	4.4	22.36	3.54	1.57	0.04
	**FFMQ tot**	**0.86**	**131.03**	**16.72**	**123.13**	**11.38**	6.96 *	**0.16**
HADS	Anxiety	0.76	9.31	2.91	9.77	3.19	0.90	0.02
	**Depression**	**0.82**	**8.13**	**1.96**	**9.13**	**2.41**	7.26 *	**0.16**
	**HADS tot**	**0.86**	**17.44**	**4.12**	**18.90**	**4.68**	4.43 *	**0.11**
MSQ	**Sleep**	**0.75**	**14.26**	**6.05**	**15.74**	**6.04**	4.58 *	**0.11**
	Wake	0.84	13.10	5.16	13.28	5.52	0.05	0.01
	MSQ tot	0.85	27.36	10.18	29.03	10.71	1.72	0.04
rMEQ	rMEQ tot	0.51	14.74	3.53	14.13	3.81	1.21	0.03

Note. Sleep = sleep quality, Wake = daytime sleepiness. Time 0 = before lockdown, Time 1 = during lockdown. Cronbach’s *α*s were computed on the Time 0 data. An interpretable measure of effect size is reported as partial eta squared (η^2^_p_). Variables that changed significantly from Time 0 to Time 1 are reported in boldface. Significant level is indicated as follows: * *p* < 0.05; ** *p* < 0.01.

**Table 2:** SEM estimated coefficients for model 1.

Path			b	CI_lower_	CI_upper_	SE	β
**Lockdown**	**→**	**FFMQ**	−7.88 *	**−14.95**	**−1.42**	**3.45**	**−0.27**
Lockdown	→	HADS	0.85	−1.01	2.82	0.97	0.10
Lockdown	→	MSQ	0.13	−3.99	4.53	2.20	0.01
**FFMQ**	**→**	**HADS**	−0.08 *	**−0.15**	**−0.01**	**0.04**	**−0.26**
**HADS**	**→**	**MSQ**	0.82 *	**0.16**	**1.32**	**0.29**	**0.35**
FFMQ	→	MSQ	−0.04	−0.18	0.17	0.08	−0.05

Note. b = unstandardized coefficient, CI_lower_ and CI_upper_ = lower and upper 95% bootstrapped confidence intervals of b, SE = standard error, β = standardized coefficient. Significant paths are reported in boldface. Significant level is indicated as follows: * *p* < 0.05.

**Table 3:** SEM estimated coefficients for model 2.

Path			b	CI_lower_	CI_upper_	SE	β
Lockdown	→	Sleep	−0.74	−3.44	2.06	1.43	−0.06
Lockdown	→	Wake	−1.03	−3.19	0.95	1.03	−0.10
Lockdown	→	Anxiety	−0.42	−1.65	0.81	0.61	−0.07
Lockdown	→	Depression	0.76	−0.22	1.66	0.49	0.17
Lockdown	→	Observing	2.19	−0.42	4.65	1.26	0.19
**Lockdown**	**→**	**Non-judging**	−4.69 **	**−7.06**	**−2.23**	**1.24**	**−0.38**
Lockdown	→	Non-reacting	0.76	−1.30	2.61	0.99	0.09
Observing	→	Anxiety	0.01	−0.11	0.12	0.06	0.03
**Non-judging**	**→**	**Anxiety**	−0.21 **	**−0.33**	**−0.10**	**0.06**	**−0.43**
**Non-reacting**	**→**	**Anxiety**	−0.15 *	**−0.28**	**−0.01**	**0.07**	**−0.21**
Observing	→	Depression	−0.01	−0.13	0.08	0.05	−0.04
Non-judging	→	Depression	−0.07	−0.16	0.03	0.05	−0.19
**Non-reacting**	**→**	**Depression**	−0.12 *	**−0.26**	**−0.01**	**0.07**	**−0.23**
**Observing**	**→**	**Sleep**	0.21 *	**0.04**	**0.47**	**0.11**	**0.20**
**Non-judging**	**→**	**Sleep**	−0.29 *	**−0.61**	**−0.02**	**0.14**	**−0.30**
Non-reacting	→	Sleep	0.19	−0.15	0.61	0.19	0.14
Observing	→	Wake	0.12	−0.05	0.33	0.09	0.13
Non-judging	→	Wake	−0.11	−0.35	0.10	0.11	−0.12
Non-reacting	→	Wake	0.04	−0.32	0.39	0.18	0.04
**Anxiety**	**→**	**Sleep**	0.55 *	**0.08**	**1.13**	**0.27**	**0.28**
Depression	→	Sleep	−0.03	−0.74	0.60	0.35	−0.01
**Anxiety**	**→**	**Wake**	0.81 *	**0.40**	**1.16**	**0.20**	**0.47**
Depression	→	Wake	−0.05	−0.57	0.47	0.26	−0.02

Note. b = unstandardized coefficient, CI_lower_ and CI_upper_ = lower and upper 95% bootstrapped confidence intervals of b, SE = standard error, β = standardized coefficient. Significant paths are reported in boldface. Significant level is indicated as follows: * *p* < 0.05, ** *p* < 0.01.

## Data Availability

The data presented in this study are available in Table S1.

## Supplementary Materials

The following are available online at https://www.mdpi.com/article/10.3390/ijerph182413149/s1, Table S1: MirolliEtAl_IJERPH_SupplementaryMaterialS1_Data.csv.
